# Wind farm development on peatlands increases fluvial macronutrient loading

**DOI:** 10.1007/s13280-019-01200-2

**Published:** 2019-05-28

**Authors:** Kate Heal, Antony Phin, Susan Waldron, Hugh Flowers, Patricia Bruneau, Andrew Coupar, Alan Cundill

**Affiliations:** 1 School of GeoSciences, University of Edinburgh, Crew Building, Alexander Crum Brown Road, Edinburgh, EH9 3FF UK; 2 Department of Geographical and Earth Sciences, University of Glasgow, Glasgow, G12 8QQ UK; 3 School of Chemistry, University of Glasgow, Glasgow, G12 8QQ UK; 4 Scottish Natural Heritage, Silvan House 3rd Floor, 231 Corstorphine Road, Edinburgh, EH12 7AT UK; 5 Scottish Natural Heritage, The Links, Golspie Business Park, Golspie, Sutherland KW10 6UB UK; 6 Scottish Environment Protection Agency, Inverdee House, Baxter Street, Aberdeen, AB11 9QA UK; 7 CampbellReith, Friars Bridge Court, 41-45 Blackfriars Road, London, SE1 8NZ UK

**Keywords:** Carbon, Land-use change, Nitrogen, Peat, Phosphorus, Upland rivers

## Abstract

Wind farms can help to mitigate increasing atmospheric carbon (C) emissions. However, disturbance caused by wind farm development must not have lasting deleterious impacts on landscape C sequestration. To understand the effects of wind farm development on peatlands, we monitored streamwater at Europe’s second largest onshore wind farm (539 MW), Whitelee, Scotland, for 31 months. Using nested catchment sampling to understand impacts on water quality, increasing macronutrient concentrations and exports were associated with wind farm development, particularly forest-felling and borrow pits. Low/poor water quality occurred in small headwater catchments most disturbed by development. At the site exit, dissolved organic C and soluble reactive phosphorus (SRP) concentrations increased during construction, though [SRP] recovery occurred within 2 years. Since C was lost and streamwater quality negatively affected, we propose future good practice measures for wind farm development, including limiting total disturbance within individual catchments and locating borrow pits, where deemed necessary, off site avoiding peatlands.

**Electronic supplementary material:**

The online version of this article (10.1007/s13280-019-01200-2) contains supplementary material, which is available to authorised users.

## Introduction

Organic soils, such as peat, cover ~ 3% of the global land mass, and are particularly important in Scotland (UK), constituting almost a quarter of Scotland’s land area (Chapman et al. 2009). Onshore wind capacity in Scotland currently exceeds all other renewable energy resources combined (Scottish Government 2017). In October 2017, Scotland had 3274 operational onshore wind turbines, 1515 under or awaiting construction, and more than 820 awaiting planning consent (Scottish Government 2017). Many wind farms are located on peatlands because these sites are typically windy, remote, and generate low returns from agriculture and other land uses. A locational analysis of Scottish wind farms estimated that 74% of ≥ 50 MW wind farms are on shallow-to-medium depth (< 1 m) and deep (> 1 m) peat (Waldron et al. 2015). However, UK peatlands generate greenhouse gas emissions of 3.72 Mt CO_2_ eq year^−1^, with many peatlands finely balanced between being a C source or sink (Worrall et al. 2011). Thus, this ecoregion must be managed to maximise carbon storage and support other ecosystem services such as water source protection and flow regulation.

The Scottish Government’s carbon calculator tool (Smith et al. 2011) can be used at pre-planning stage to identify if landscape disturbances from a wind farm development are offset by reduced C emissions. However, C loss through drainage remains uncertain (Nayak et al. 2010) as few studies have assessed this. The main activities of wind farm development are track construction, quarried aggregate extraction (“borrow pits”) and turbine foundation excavation. Furthermore, since afforestation of UK peatlands is widespread (Hargreaves et al. 2003), felling is often undertaken to create space and increase turbine wind yield. Removing peat and surface vegetation affects C sequestration: the impacts may continue until recovery or restoration of the site occurs. Fluvial export of macronutrients other than C, such as phosphorus (P) and nitrogen (N), may also increase with soil and vegetation disturbance.

Landscape disturbance for wind farm construction can be intense and spatially variable. To understand this impact from terrestrial observations would be too challenging for the high-resolution spatial coverage required. However, catchment hydrochemistry integrates the response to different processes within the landscape: numerous studies have sampled water to understand the influence of land use and management on water chemistry concentrations and exports, using longitudinal sampling along a watercourse or at the outlets of paired or nested sub-catchments with a variety of land uses (e.g. Tetzlaff et al. 2007; Löfgren et al. 2009). Further, from long-term monitoring of surface waters (internationally) we understand what is typical, and good, water quality for a region and so can assess if water quality has changed. Both longitudinal and long-term streamwater sampling approaches were used in this study to understand the effects of landscape disturbance on the macronutrients P and N as increased mobilisation from soils and vegetation modified by wind farm construction may potentially result in eutrophication of surface waters (e.g. Riscassi and Scanlon 2009).

Our 10-year fluvial macronutrient monitoring programme at the outlets of catchments draining the 539 MW Whitelee wind farm, the largest onshore wind farm in the UK and the second largest in Europe by generating capacity (EWEA 2013), indicated short-term increases in river water macronutrient concentrations (Zheng et al. 2018) and export following construction (Murray 2012). For example, [SRP] increased towards the end of and after phase 1 development in 2009 (Murray 2012, Fig. 1). Thus, when the wind farm expanded in phase 2 starting in 2010, we undertook research within the catchment most affected by construction to understand to what extent wind farm development could lead to changes in water quality. We wanted to assess, if there is an impact on water quality (by changes in macronutrient concentration): (i) which types of development disturbance have impact, and (ii) how long until river water is impacted, and subsequently recovers?

**Fig. 1:**
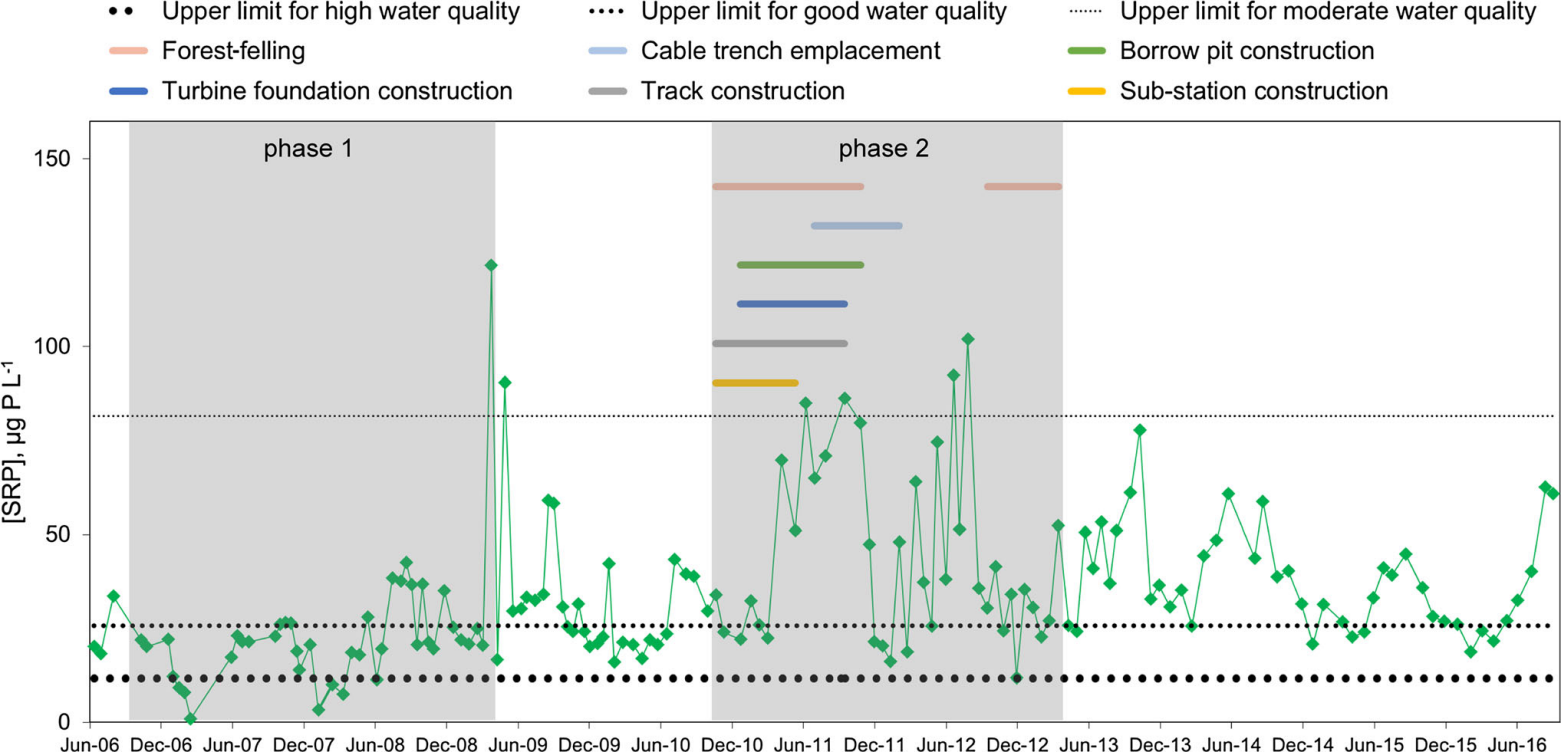
Soluble reactive phosphorus (SRP) concentrations measured at the outlet of catchment WL15 (sampling point 15_1, see Fig. 2a for location), July 2006–September 2016 (based on Zheng 2018). Dotted lines represent site-specific annual mean RP standard thresholds (UKTAG 2013). The boxes show the timing of phases 1–2 of wind farm development, whilst the colour bars depict the timing of different activities during phase 2

## Materials and methods

### Site description

**Fig. 2:**
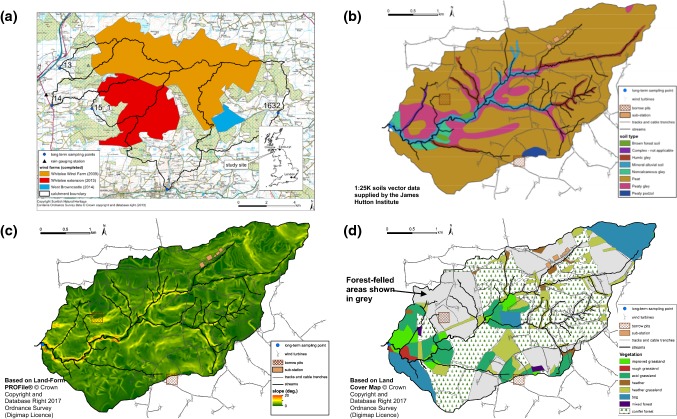
**a** Long-term streamwater sampling points, wind farm development and catchment locations; WL15 catchment maps of **b** soil types, **c** slope and **d** vegetation, overlain by wind farm extension activities

Whitelee wind farm (539 MW) is located on Eaglesham Moor (55°40′24 N, 4°16′00 W), 16 km south of Glasgow, central Scotland (Fig. 2a). Development of the wind farm started in 2006 and occurred in two main phases: 140 turbines in phase 1 by 2009 and phase 2 (75 turbines) constructed from November 2010 to March 2013.

The wind farm is located on a plateau with a maximum elevation of 376 m. Mean annual rainfall is 1342 mm (1975–2002); the lowest and highest annual mean air temperatures are 4.2 °C and 11.5 °C (1998–2005) (Murray 2012; M. Chalton pers. comm.). The underlying geology comprises Carboniferous porphyritic basalts and members of the calciferous sandstone series. The bedrock is mostly overlain with ~ 3 m of glacial and recent drift deposits and peat, predominantly as blanket bog. The mean peat depth, measured at 161 locations in phase 1, was 1.90 m (SD ± 1.35 m) (CRE Energy 2002).

The main land use in the area has been Sitka spruce-dominated (*Picea sitchensis*) forestry plantation established in the 1960s–1970s. Large forest tracts were felled for wind farm construction to clear land for building turbine foundations (~ 3000 m^2^ surface area disturbance per turbine, Waldron et al. 2018), and to reduce surface roughness that decreases power outputs. Other significant wind farm development activities included borrow pit excavation followed by restoration; turbine foundation excavation; adjacent hardstanding for turbine maintenance; substations; and tracks for access and to carry cabling alongside. Existing forestry tracks were upgraded and new tracks constructed using stone from borrow pits or as floating tracks over peat > 1 m depth and gradients of < 1:10. To mitigate the effects of disturbance, tracks were routed to avoid sensitive areas and silt fences and settling ponds used to manage suspended solids in runoff.

A long-term monitoring programme of Whitelee wind farm catchment macronutrient concentrations commenced in July 2006 (Waldron et al. 2009) and continued until September 2016. Of the long-term monitoring catchments, the WL15 catchment was selected for this investigation due to the high percentage of the catchment area affected by the extension (see Fig. 2a), hosting 31 of the 75 turbines.

The 11.45 km^2^ WL15 catchment is drained by a small third-order river, the Hareshawmuir Water. Blanket peat (> 0.5-m-depth organic horizon) covers most of the catchment, with gleys and mineral alluvial soils occurring adjacent to river channels particularly in the lower reaches (Fig. 2b). Elevation is 180–330 m and the topography is relatively homogeneous, with slopes typically < 3°, but steeper in the vicinity of river channels (Fig. 2c). 3.8 km^2^ of the 70% conifer forest plantation cover in the catchment was felled in stages in 2006–2013, predominantly due to the wind farm development but also for timber harvesting. Brash was left as mats in the felled areas to protect soils from heavy machinery, followed by establishment of grasses and rushes. Other than forest plantation, vegetation cover is mainly grassland and bog. Annual rainfall was 1823 and 1252 mm for hydrological years 2012 (HY2012, 1 October 2011–30 September 2012) and 2013 (HY2013, 1 October 2012–30 September 2013), respectively (data from SEPA tipping bucket rain gauge at Amlaird, ~ 4 km west). For the 10-year 2009–2018 Amlaird dataset, the study year rainfall totals are ranked the 1st and 7th highest.

**Fig. 3:**
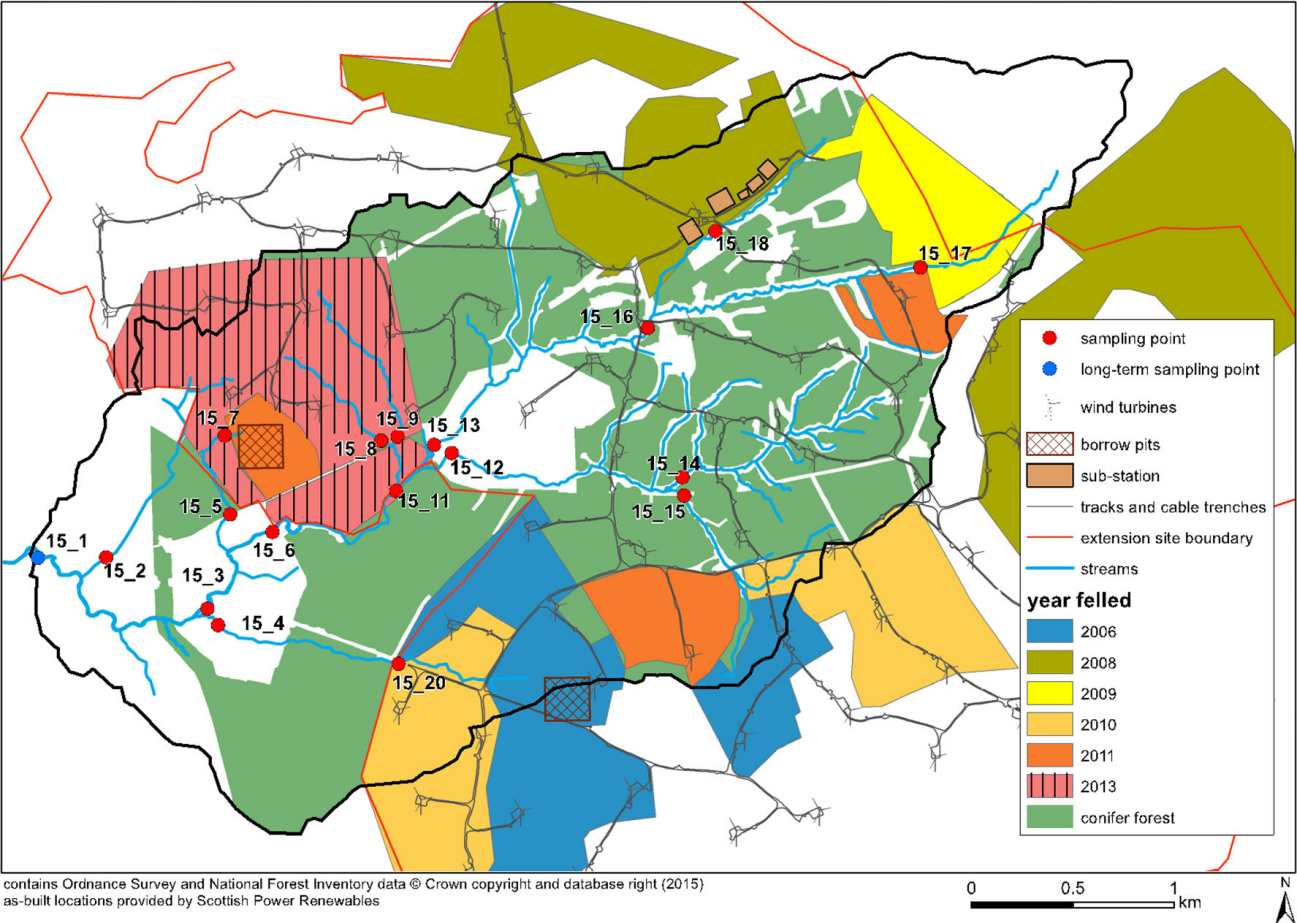
Streamwater sampling points in the WL15 catchment overlain on wind farm extension activities. The solid black line depicts the catchment boundary. Boundaries of sub-catchments draining to streamwater sampling points are not shown

A spatially nested streamwater sampling programme was designed to target different wind farm development activities (Fig. 3). Given the relatively homogeneous nature and small scale of the catchment, macronutrient concentrations are hypothesised to be similar at all streamwater sampling points, with any differences attributable to spatial differences in land use and human activities. Key characteristics of the sub-catchment area draining to each sampling point are shown in Table 1. Samples were collected every 3 weeks from October 2011 to March 2014 at 18 locations across the WL15 catchment, including the long-term monitoring point at 15_1. This sampling frequency captured samples from a full range of river flows (Fig. S1). At 15_18, sampling ceased in May 2013 because of changed hydrological conditions as the result of drainage diversion. Sampling across the catchment was carried out within ~ 7 h and all sub-catchments were within an area of ~ 12 km^2^. Thus, temperature and rainfall can be assumed comparable for all sampling points.

**Table 1:** Key characteristics of the sub-catchment area draining to each streamwater sampling point

Sampling point	Nested catchments	Catchment area (km^2^)	Peat (%)	Forest-felled (%)	Forest plantation (%)	Grassland and bog (%)	Turbines (no.)	Turbine density (no. km^−2^)	Track length (km)	Track density (km km^−2^)	Borrow pit (%)	Distance to nearest disturbance (km)
*15_1*	*All*	*11.45*	*78*	*33*	*37*	*30*	*31*	*2.7*	*44*	*3.8*	*0.5*	*1.88*
15_2*	**None**	**0.41**	**71**	**45**	**20**	**36**	**0**	**0**	**0.1**	**0.2**	**0**	**0.77**
*15_3*	*All bar 15_1, 15_2*	*8.66*	*79*	*34*	*39*	*27*	*28*	*3.6*	*39*	*4.5*	*0.5*	*0.68*
15_4*	15_20	0.76	85	67	24	9	2	2.6	4.6	6.0	1.2	0.91
15_5*	15_7	0.16	82	79	12	9	1	6.2	0.3	1.9	13.5	0.22
*15_6*	*15_8*-*15_18*	*8.19*	*81*	*35*	*39*	*26*	*27*	*3.3*	*39*	*4.7*	*0.3*	*0.07*
15_7*	**None**	**0.03**	**100**	**99**	**1**	**0**	**1**	**33**	**0.3**	**9.4**	**0**	**0.00**
15_8*	**None**	**0.26**	**73**	**100**	**0**	**0**	**1**	**3.8**	**1.5**	**5.6**	**0**	**0.00**
15_9*	**None**	**0.53**	**82**	**65**	**29**	**5**	**4**	**7.5**	**3.4**	**6.4**	**0**	**0.00**
*15_11*	*15_8, 15_9, 15_12*-*15_18*	*7.64*	*83*	*33*	*39*	*27*	*27*	*3.5*	*38*	*5.0*	*0*	*0.00*
15_12*	15_14, 15_15	2.69	80	22	57	21	12	4.5	17	6.3	0	0.50
15_13*	*15_16*-*15_18*	*3.92*	*86*	*31*	*31*	*38*	*10*	*2.6*	*16*	*4.2*	*0*	*1.31*
15_14*	**None**	**0.82**	**86**	**1**	**77**	**22**	**4**	**4.9**	**6.2**	**7.5**	**0**	**0.21**
15_15*	**None**	**0.83**	**86**	**47**	**48**	**5**	**4**	**4.8**	**5.4**	**6.6**	**0**	**0.43**
*15_16*	*15_17, 15_18*	*2.70*	*92*	*39*	*20*	*41*	*5*	*1.8*	*9.0*	*3.3*	*0*	*0.00*
15_17*	**None**	**0.50**	**87**	**36**	**0**	**64**	**1**	**2.0**	**0.3**	**0.6**	**0**	**0.00**
15_18*	**None**	**1.16**	**99**	**37**	**16**	**48**	**1**	**0.9**	**2.3**	**2.0**	**0**	**0.00**
15_20*	**None**	**0.41**	**93**	**96**	**4**	**0**	**2**	**4.9**	**3.7**	**9.0**	**2.2**	**0.00**

Independent sampling points are shown in bold. Italics denote sampling points on the main river stem

* indicates sub-catchments included in regression analysis (see text for explanation). % refers to the % sub-catchment area covered by peat/affected by different activities. Some % totals for forest-felled/forest plantation/grassland and bog do not equal 100% due to rounding

Methods for water sample collection and analysis and quality control procedures are detailed in Supplementary Material S1. Briefly, dissolved organic carbon (DOC) was quantified on acidified, degassed filtrate (Whatman^®^ GF/F 0.7 μm filter size, as in many other studies, e.g. Dyson et al. 2011) using a Thermolux total carbon analyser. Particulate organic carbon (POC) was determined by loss-on-ignition of the residue from sample filtration.

Soluble reactive phosphorus (SRP) and total oxidised nitrogen (TON, NO_3_^−^ plus NO_2_^−^) concentrations were determined by colorimetric methods using a Bran + Luebbe^®^ Autoanalyzer 3, usually within 24 h of sample collection, on 0.2 μm nylon membrane-filtered samples to maximise sample stability prior to analysis (Worsfold et al. 2016). Limits of detection were 6.3 µg P L^−1^ and 0.08 mg N L^−1^.

Alkalinity was determined using manual Gran titration with 0.01 M HCl. The mean alkalinity for each sampling site was used to calculate site-specific annual mean reactive [P] for the lower-class boundaries of high, good, moderate and poor ecological status as described in UKTAG (2013). The impact of land-use change was assessed by comparing annual mean river water [SRP] with these EU Water Framework Directive (WFD) Environmental Quality Standards (EQS).

### Flow and export estimates

Flow and export estimate procedures are detailed in Supplementary Material S2. Flows were measured at four representative locations within the catchment (Fig. S2) to build relationships with flow at a SEPA gauging station ~ 6 km south. A continuous estimate of flow at each sampling point during the study period was derived using these relationships scaled for sub-catchment area. Mass exports of DOC, POC, SRP and TON were estimated for all streamwater sampling locations using mean daily flow and 3-weekly concentration data. An interpolation method (see S2) was used since there were no significant relationships between concentrations and flow. P and N exports were calculated on the major macronutrient pools (SRP and TON) and do not include organic P and N which, if included, would increase the export quantities (e.g. Chapman et al. 2001; Kortelainen et al. 2006). We aimed to assess how water quality may be affected and thus focussed on the nutrient classification used in water quality standards.

### GIS and data analysis

Digital terrain model, soil type and drainage, vegetation and land-use datasets (detailed in Supplementary Material S2) were entered into a Geographic Information System (ArcMap™ v.10) and used with delineated sub-catchment boundaries to generate catchment descriptors (Tables 1 and S2). These descriptors were used in multiple linear regression analysis (MLRA) to quantify how wind farm development and catchment physiography control macronutrient export and streamwater concentrations, after checking for autocorrelation between variables. The total annual export per unit area and median annual DOC, POC, SRP and TON concentration for each sub-catchment were response variables. Separate analyses were conducted for HY2012 and HY2013 to consider the timing of wind farm development controls.

The rationale for sub-catchment selection and MLRA procedures are detailed in Supplementary Material S3. Briefly, 13 sub-catchments (the nine independent headwater catchments and four downstream sampling points) were selected for the HY2012 MLRA to maximise the use of the data collected whilst minimising the influence of nested sampling points. The number of sub-catchments was reduced to 12 for the HY2013 MLRA due to cessation of sampling at 15_18 as explained above. As the maximum number of variables in MLRA should be at least one fewer than the number of response variables, process understanding informed our selection of variables from Tables 1 and S2 to include in MLRA. The 10 predictor variables selected and the rationale for their selection are detailed in Table 2; five were expected catchment physiographic controls on streamwater macronutrient concentration and export and the remainder reflected wind farm construction activities.

**Table 2:** The predictor variables used in the multiple linear regression analyses and their rationale for consideration. All predictor variables were used for HY2012 and HY2013, apart from forest-felling (total) which was used only in models for HY2013

Predictor variable	Rationale
Soil types (proportion catchment area)	Carbon-rich soils such as peat have been observed to (i) have a positive control on [DOC] in streamwater (Temnerud and Bishop 2005), (ii) have a negative control on nitrate concentrations (Cundill et al. 2007), and (iii) not retain P (Cummins and Farrell 2003a), implying a positive control on [SRP]. Soil drainage classes were autocorrelated with soil type and therefore were not included in this analysis. Peaty gley and podzol were autocorrelated with peat so proportion peat was used to represent soil type as the dominant soil
Mean slope (º)	Calculated as mean of slopes in all 1 m^2^ grid cells in each catchment. It should be positively related with runoff generation and negatively related with catchment water residence time
Drainage density (km km^−2^)	The greater the drainage density, the more connected the drainage networks will be with the soil. Streamwater residence time and the drainage density will likely be negatively related, and both are also linked to slope
Forest, grassland and bog (proportion catchment area)	Forest plantation changes soil drainage and runoff. Tree needles can also scavenge atmospheric deposition and therefore existing forest must also be considered. Non-forested areas (grassland and bog) consist mainly of acid and heather grassland or very boggy areas, and tend to be adjacent to river channels that were not afforested during planting in the 1960s–1970s. These are weakly autocorrelated with track density, but are included as they have historically not been subject to land-use change, unlike the rest of the catchment
Forest-felling (total) (proportion catchment area)	The total deforestation in each catchment across wind farm development phases 1–2. Since felling was not completed until 2013, this variable was not entered in the models for HY2012. Machinery used for forest-felling and stump removal can cause soil disturbance, exposing deeper soil to respiration. Forest-felling debris represents a source of carbon and nutrients when left in situ. Forest-felling is therefore observed to have a positive relationship with carbon and nutrients in streams (e.g. Cummins and Farrell 2003a, b)
Forest-felling > 1 year (proportion catchment area)	Since forest-felling was ongoing, in the HY2012 models only felling that had occurred > 1 year ago was considered. In the HY2013 models both felling > 1 year and total forest-felling were entered as predictor variables to take account of the recent felling and also to differentiate between the two main periods of forest-felling during the wind farm development in phase 1 and phase 2, respectively
Borrow pits (proportion catchment area)	Borrow pits involve excavating significant volumes of surface soil and vegetation and subsequently rock for aggregate, followed by infilling with peat/soil and restoration. These activities may affect macronutrient sources and mobility and flow pathways. Although weakly autocorrelated with forest-felling > 1 year, borrow pits were included to represent all aspects of wind farm construction activity
Track density (km km^−2^) and turbine density (n km^−2^), and their combined effect	Tracks allow access and connect turbines and electricity cabling generally runs alongside tracks underground. Tracks can be floating or cut into the land surface depending on soil type. Turbines are regularly spaced but the track or turbine density may not be constant, depending on catchment characteristics and so both controls need to be considered, as well as the combined effect of both
Distance from streamwater sampling point to nearest disturbance (km)	The distance to the nearest disturbance, whether tracks, turbine bases, the substation, borrow pits or forest-felling. There could be natural attenuation of macronutrient loading during hydrological flow paths before entering and within the river system and so this measure of distance accommodates that capacity

Three sets of MLRA were conducted to ensure that all possible dimensions of wind farm infrastructure were considered (see Supplementary Material S3 for details). This generated several regression models of the significant controls on macronutrient export and concentration, which have strong similarities and some differences. Where a control was consistently identified as significant across the models, we could be confident it influenced either macronutrient concentration or export; if it only appeared in one model, then less so. In the results, we present for simplicity the detailed output of the model which included a combined wind farm infrastructure disturbance variable, but all model outputs are summarised in Table S3.

Statistical analyses were conducted in Minitab^®^ v.18, with significance defined as *p* < 0.05. All datasets analysed were tested for normality using Anderson–Darling tests and transformed if required.

## Results

### Impact of wind farm development on river water macronutrient concentrations and receiving aquatic systems

Table 3 shows summary statistics for streamwater macronutrient concentrations. Median streamwater [DOC] was high and variable across the sub-catchments, ranging from 21.6 in 15_18 to ~ 60 mg C L^−1^ in 15_5 and 15_7. Minimum and maximum [DOC] were typically 6–10 and 50–70 mg C L^−1^, respectively. Median [POC] ranged from 0.82 to 2.62 mg C L^−1^ and accounted for only 2.2–6.9% of median total OC concentrations ([DOC] + [POC]) in the sub-catchments. [TON] was generally very low as expected for these upland headwater streams, with median [TON] of 0.02 to 0.32 mg N L^−1^. Maximum [TON] was typically 0.2–0.3 mg N L^−1^, although higher maximum concentrations up to 1.60 mg N L^−1^ were measured in 15_3, 15_4 and 15_20.

**Table 3:** Summary statistics of measured streamwater macronutrient concentrations October 2011–March 2014. LOD = limit of detection. n is lower at 15_18 as sampling ceased in May 2013 due to drainage diversion

Sub-catchment	DOC (mg C L^−1^)				POC (mg C L^−1^)				SRP (µg P L^−1^)					TON (mg N L^−1^)			
	Median	Min	Max	*n*	Median	Min	Max	*n*	Mean	Median	Min	Max	*n*	Median	Min	Max	*n*
15_1	27.8	8.9	56.8	41	1.05	0.04	6.90	41	41	36	12	102	40	0.09	< LOD	0.32	41
15_2	35.8	8.6	66.8	38	0.82	0.00	5.16	41	34	29	4	96	41	0.03	< LOD	0.33	41
15_3	29.0	10.6	62.8	41	1.39	0.12	18.0	41	52	39	9	213	41	0.09	< LOD	0.68	41
15_4	30.8	9.1	62.4	41	1.78	0.18	9.57	41	84	76	21	210	41	0.29	< LOD	1.60	40
15_5	57.6	17.1	137	41	2.00	0.12	47.3	41	132	69	9	496	41	0.05	< LOD	0.25	41
15_6	27.2	9.5	54.6	40	1.60	0.12	12.0	41	41	39	8	96	41	0.09	< LOD	0.20	41
15_7	61.1	26.9	114	39	2.62	0.03	15.2	36	378	327	155	1034	38	0.08	< LOD	0.32	39
15_8	36.1	16.8	87.0	39	2.29	0.11	26.3	41	102	83	13	356	41	0.02	< LOD	0.27	40
15_9	33.4	10.7	79.7	40	1.32	0.16	9.37	41	48	45	12	126	41	0.05	< LOD	0.25	40
15_11	28.2	10.2	54.8	41	1.15	0.04	4.88	41	46	38	13	97	41	0.09	< LOD	0.19	41
15_12	29.5	10.7	59.6	41	1.62	0.02	5.88	41	57	52	18	120	41	0.11	< LOD	0.21	41
15_13	23.9	8.4	49.6	38	1.16	0.15	3.88	41	35	30	7	82	41	0.08	< LOD	0.23	41
15_14	26.2	9.1	66.2	38	1.26	0.04	3.25	41	37	33	13	90	41	0.09	< LOD	0.21	41
15_15	37.6	9.6	68.0	41	2.03	0.22	12.4	41	74	63	24	179	41	0.14	< LOD	0.28	41
15_16	23.5	7.6	47.0	41	1.30	0.02	6.08	41	34	33	3	89	41	0.08	< LOD	0.25	41
15_17	25.9	6.1	49.1	41	1.33	0.02	4.89	41	35	34	9	78	41	0.08	< LOD	0.25	41
15_18	21.6	6.4	49.1	28	1.12	0.02	9.85	28	29	27	2	70	27	0.08	< LOD	0.20	28
15_20	34.0	10.5	69.4	41	2.53	0.11	8.62	41	117	91	37	274	41	0.32	0.09	1.05	40

Median [SRP] ranged from 27 in 15_18 to 91 µg P L^−1^ in 15_20, with a considerably higher value of 327 µg P L^−1^ in 15_7, the smallest sub-catchment almost entirely affected by forest-felling. From comparing mean [SRP] (Table 3) with EU WFD EQS set to protect aquatic ecosystems from eutrophication (UKTAG 2013), 12 of the 18 sub-catchments would be classified as “moderate”, five as “poor” and one as “bad” status. During development of the wind farm extension and the following summer 2012, [SRP] river water status at the catchment exit (15_1) deteriorated from “good”/“moderate” to “moderate”/”poor” (Fig. 1). However, for the remaining four years of the 10-year outlet monitoring programme, the status returned to “good”/“moderate” although mostly “moderate”.

**Fig. 4:**
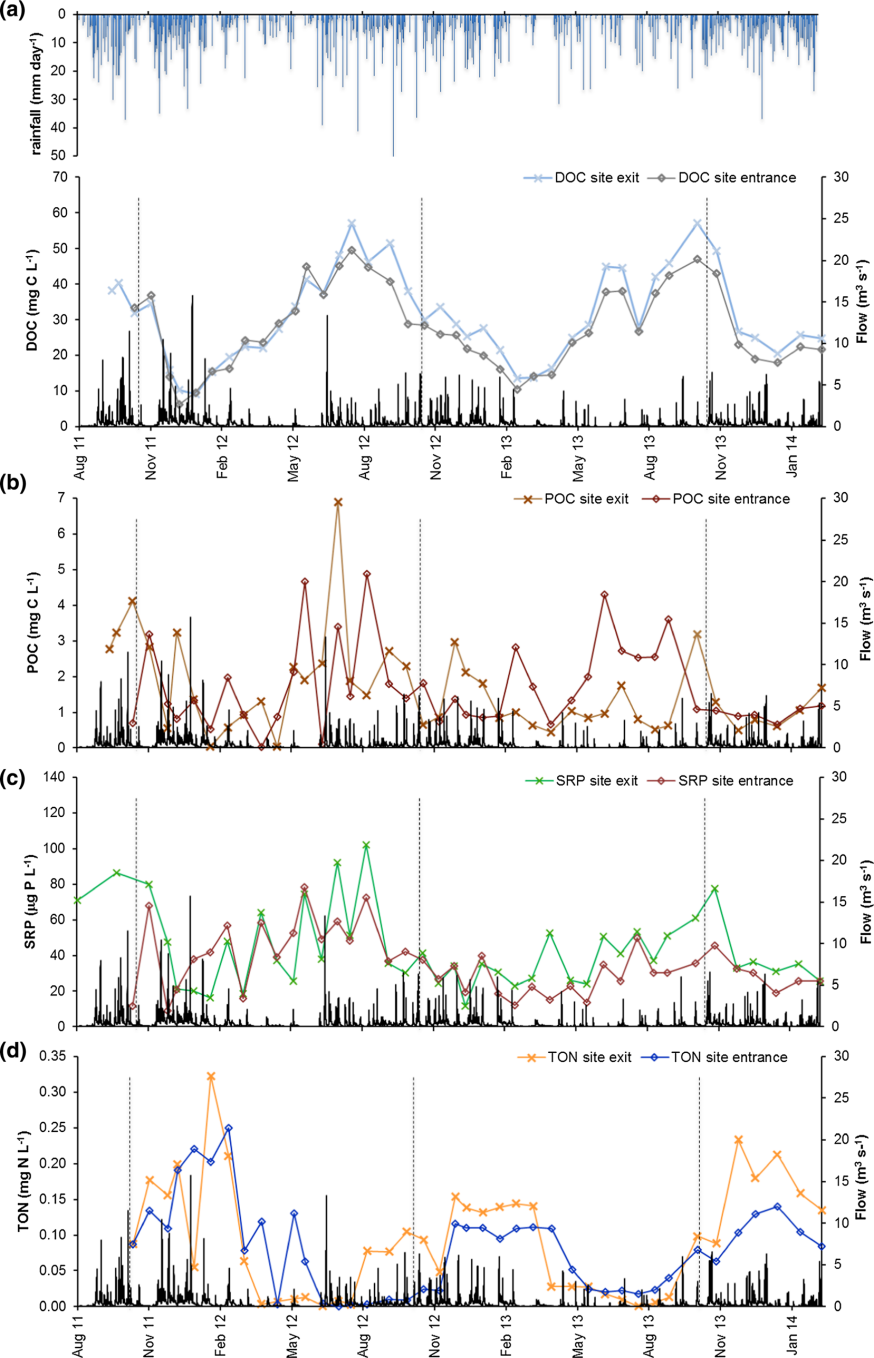
Time series of **a** [DOC], **b** [POC], **c** [SRP] and **d** [TON] August 2011 to March 2014 at the wind farm extension site entrance (furthest upstream sampling point at 15_17) and exit (15_1, downstream of extension activities). Hydrological years are marked by dashed lines. Rainfall data are from the rain gauge at Amlaird; flow is at the site exit (15_1)

To determine whether there was an overall impact of wind farm extension construction activities, macronutrient concentrations were compared for the whole sampling period (August 2011–March 2014) between the extension entrance (furthest upstream sampling point at 15_17) and exit (15_1, downstream of extension activities) (Fig. 4). Significant differences (paired t tests) were identified between the site entrance and exit for [DOC], [SRP] and [TON] in which the mean concentration was higher at the exit than the entrance. [DOC] and [SRP] differed significantly between the site entrance and exit for the entire period ([DOC]: *n* = 41, difference = 3.04 mg C L^−1^, *p* < 0.001; [SRP]: *n* = 40, difference = 5.8 μg P L^−1^, p < 0.05). Since a lag between land-use change and [DOC] response has been shown in other studies (Muller et al. 2015), often attributed to catchment drying and re-wetting, the macronutrient time series were analysed separately before and after mid-July 2012 when a period of low flow ended. For all macronutrients, no significant differences in concentrations were detected in the period October 2011 to mid-July 2012. However, after this date significant differences appeared in [DOC], [SRP] and [TON] between the wind farm entrance and exit ([DOC]: *n* = 27, difference = 4.58 mg C L^−1^, *p* < 0.001; [SRP]: *n* = 27, difference = 8.7 μg P L^−1^, *p* < 0.001; [TON]: *n* = 27, difference = 0.028 mg N L^−1^, *p* < 0.01).

### Spatial variation in macronutrient exports and sources in relation to disturbance associated with wind farm development

**Fig. 5:**
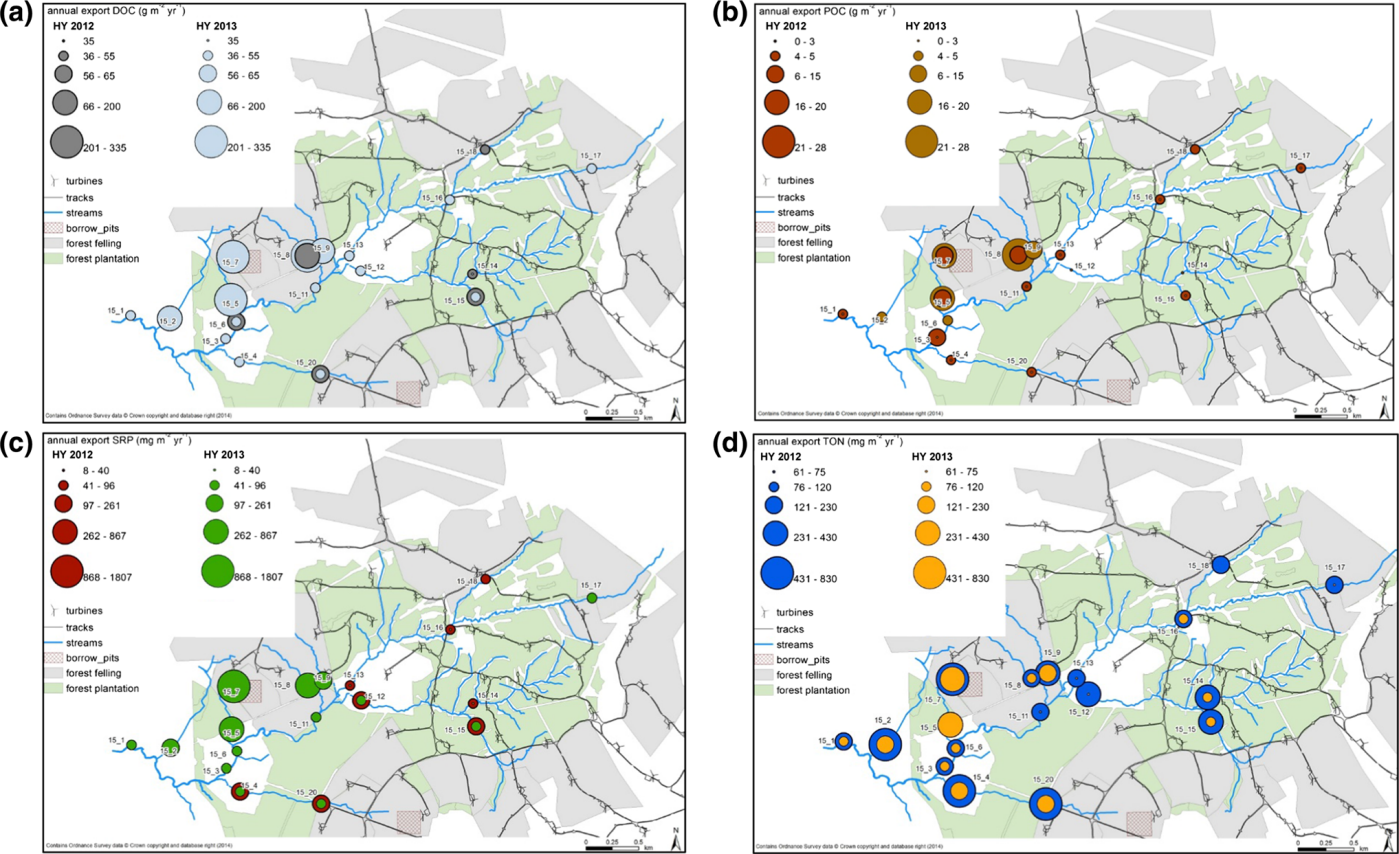
Annual macronutrient export (**a** DOC, **b** POC, **c** SRP, **d** TON) at WL15 sub-catchment outlets overlain for HY2012 and HY2013. Where both years cannot be seen, the exports were in the same range. For sub-catchment 15_18, exports are shown for 2012 only

Area-normalised DOC, POC, SRP and TON exports in HY2012 and HY2013 are shown for each sub-catchment sampling point in Fig. 5. Export rates were similar at the entrance (15_17) and exit (15_1) of the extension site. DOC, POC and SRP exports had similar spatial patterns across the WL15 catchment, which contrasted with TON exports. At headwater sites impacted by forest-felling (15_5, 15_7, 15_8, 15_9) DOC, POC and SRP exports were substantially higher than in the Hareshawmuir Water main stem. TON exports were also high at these locations and in sub-catchments 15_2, 15_4 and 15_20. The spatial pattern of DOC and SRP exports was similar in both HYs in many sub-catchments (depicted by just one circle colour in Fig. 5). Higher POC and TON exports occurred in some sub-catchments in HY2012 (the wetter year, annual rainfall 1823 mm) compared to HY2013.

### Controls on macronutrient concentrations and exports in sub-catchments

Multiple linear regression analysis (MLRA) of controls on fluvial macronutrient export and median concentration in HY2012 and HY2013 (Table 4, Table S3) captured variations between sub-catchments well (adjusted *R*^2^ 70.8–91.1%, median 82.6%), except for TON export, although HY2013 (adjusted *R*^2^ 51.4%) was better explained than HY2012 (adjusted *R*^2^ 17.4%).

**Table 4:**
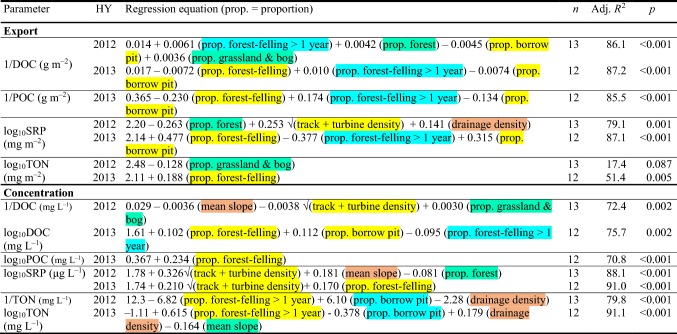
Controls on annual exports and median concentrations of DOC, POC, SRP and TON for HY2012 and HY2013 for selected sub-catchments. For POC, equations are shown for HY2013 data only due to the possible under-estimation of [POC] in HY2012 (see Supplementary Material S1). Terms in regression equations are all significant (*p* < 0.05), have standardised coefficients and are ordered in decreasing order of significance. % *R*^2^ (adj) accounted for by each variable in the MLRA is shown in Supplementary Material Table S3. Text shading highlights positive and negative catchment and wind farm development controls:  . In reciprocal transformations (1/*x*), the sign on the model term has the reverse influence. Thus, a negative term has a positive influence on *x* and a positive term has a negative influence on *x*

Adjusted *R*^2^% for each significant control varied between individual MLRA equations (Table S3). Catchment physiographic controls with higher adjusted *R*^2^ occurred in equations modelling macronutrient export in HY2012; they were proportion forest (16.9 and 26.1% for DOC and SRP exports, respectively) and proportion grassland and bog (17.3 and 17.4% for DOC and TON exports, respectively). Drainage density had a lower adjusted *R*^2^ (10.7%) in the HY2012 [TON] model. Adjusted *R*^2^ for wind farm construction controls were generally higher, but also varied more between MLRA equations. Proportion forest-felled had values of 23.4% for HY2013 DOC export rising to 51.4–57.0% for POC, SRP and TON HY2013 export and 70.8% for HY2013 [POC]. Adjusted *R*^2^ for proportion forest-felled > 1 year were 9–10% for HY2013 POC and SRP export, increased to ~ 20–30% for DOC exports, HY2012 [DOC] and [TON], and was 45.5% for HY2013 [TON]. Proportion borrow pit accounted for most variability in DOC export (30.0% in HY2012 and 35.2% in HY2013) and HY2012 [TON] (50.3%), but also accounted for 20–30% of the variability in HY2013 SRP export and HY2013 [TON]. The combined track density + turbine density control was significant mainly for SRP export and concentration, accounting for 40–70% of variability in HY2012 SRP export and [SRP] in both years.

A combination of catchment physiographic characteristics and wind farm development activities significantly influenced all macronutrient exports and concentrations in HY2012, apart from TON export where the only significant control was proportion grassland and bog (Table 4, Table S3). However, in HY2013 only wind farm development activities were significant controls on all macronutrient exports and concentrations, apart from [TON] which was controlled by both catchment physiographic characteristics and wind farm development activities. Five of the eight significant controls identified in the MLRA had a consistent positive or a negative influence; e.g. drainage density, where significant, always had a positive influence on macronutrient exports and concentrations, whilst proportion forest-felled always had a negative influence. The exceptions were the three controls—mean slope, proportion forest-felled > 1 year and proportion borrow pit—which had varying positive/negative influences, depending on the specific MLRA equation. Mean slope had a significant positive influence on [DOC] and [SRP] in HY2012 but a negative influence on [TON] in HY2013. Proportion forest-felled > 1 year and proportion borrow pit had the same influences, with a significant positive influence on DOC export in HY2012 and DOC, POC and SRP exports and [DOC] in HY2013, but a negative influence on [TON] in both years.

Catchment controls with the most consistent influence on macronutrient export and concentration (Table S3) were proportion forest (negative control on HY2012 DOC and SRP exports) and proportion grassland and bog (negative control on HY2012 DOC and TON exports and [DOC]). The negative controls of both these variables are interpreted as representing areas unaffected by recent disturbance from either forest-felling or wind farm construction. Soil type (proportion peat) did not commonly and consistently appear as a control, and drainage density only influenced [TON] consistently. Of the wind farm development-related controls, infrastructure density (track, turbine or combined) positively influenced only SRP export and [DOC] in HY2012, and [SRP] in both years. However, borrow pits consistently positively influenced DOC and POC export, and SRP export in HY2013, but did not influence concentration consistently, except for [TON] where there was a consistent negative influence. Proportion forest-felled was a positive control on all macronutrient exports. Proportion forest-felled > 1 year was a negative control on DOC exports in HY2012 and HY2013 and SRP export in HY2013, and a positive control on [TON] in both years.

Overall, the regressions indicated consistently that (i) an aspect of forest management (the existence of forest, its felling, time since felled) influenced the fluvial loading of all macronutrients, and (ii) wind farm infrastructure (borrow pits, track density, turbine density) influenced all macronutrient parameters except [POC]. Comparison of regression models between HY2012 and HY2013 indicated responses to wind farm development controls. For DOC, SRP and TON export, catchment-related controls such as proportion forest and grassland and bog were no longer significant in HY2013, and forest-felling and borrow pits emerged instead. Where turbine or track density appears in the models (mostly with [SRP] but also [DOC]), these have a higher explanatory power than borrow pits.

## Discussion

### Forest-felling and wind farm infrastructure as controls on fluvial macronutrient concentrations and exports

The results of MLRA (Table 4) indicate that, whilst catchment physiographic characteristics have some effect, the predominant controls on macronutrient concentrations and exports are related to the wind farm development, particularly forest-felling and borrow pits, but additionally track and turbine density. The processes underlying the significant controls are discussed below, noting that sub-catchments are not analysed individually since a consistent process response may be confounded across sub-catchments. This is because controls may be opposing in their direction of influence on macronutrients. For example, one control may affect the macronutrient source availability and/or transport, whilst another relates to buffering of macronutrients.

Forest-felling that occurred > 1 year prior to sampling negatively influences DOC export throughout the 2-year observation period and SRP export in HY2013 only. Total forest-felling proportion, which incorporated forest felled during the observation period, positively influenced all macronutrient export, and [DOC], [POC] and [SRP] in HY2013. Increased streamwater [DOC] linked with forest-felling has occurred in other catchments draining Whitelee wind farm (Zheng et al. 2018). The causes of elevated DOC and POC in sub-catchments subject to recent felling are well documented and include mobilisation from brash and exposed and disturbed soil (e.g. Nieminen et al. 2017); enhanced runoff due to reduced evapotranspiration resulting from a decrease in the number of trees (e.g. Muller et al. 2015); and an increased soil DOC pool generated by enhanced soil microbial activity stimulated by warmer soil temperatures after felling (Pérez-Batallón et al. 2001). The source of DOC is likely to come from both the brash and soil (Drinan et al. 2013; Muller et al. 2015). In this study, we are unable to determine which source is the biggest; this would require analysis of streamwater DOC composition (e.g. as conducted by Zheng et al. 2018), and/or study of forest-felling on low-DOC mineral soils.

The switch, from a negative influence of previously felled areas to a positive influence with concurrent felling, indicates greater availability of SRP and C from felled brash with limited phosphate adsorption by peat soils (Cummins and Farrell 2003a). Further, as concentration is a response for only some macronutrients and rainfall was lower in HY2013, an increase in runoff generation is necessary for a positive response in all macronutrient exports. This can occur due to decreased evapotranspiration (ET) water loss and decreased infiltration and increased surface runoff rates from a disrupted surface, both well documented with reduction in tree cover (e.g. Worrell and Hampson 1997; Marc and Robinson 2007). We do not have flow data prior to the field campaign to assess runoff change; it is a logical interpretation from the MLRA and supported by previous research (e.g. as summarised in Andréassian 2004).

The MLRA outcomes reveal that C and SRP export is reduced in areas felled prior to 2011, suggesting that initial recovery in C and SRP loss may occur already after 2 years (the response changed in HY2013). The main processes postulated to drive this response are reduced release of C and P from leaching and brash decomposition (Stevens et al. 1995) and diminishing runoff generation, due to vegetation regrowth on the felled surface resulting in increased ET (Jones and Post 2004). Only [TON] showed a positive response to previous forest-felling, suggesting a lag time > 1 year between forest-felling and TON release into streamwater. A possible explanation for this is that whilst SRP was leached rapidly from brash (as shown elsewhere, e.g. by Jamieson et al. 2018), inorganic N generation due to brash mineralisation does not occur until 1–4 years after felling (as reported for felled forests in Wales and Ireland by Stevens et al. (1995) and Asam et al. (2014), respectively). In both these studies, the inorganic N leached from brash was dominated by ammonium-N (not measured in the present study), rather than TON. However, mineralisation and nitrification of brash-derived organic N may be a possible source of TON (Stevens et al. 1995) as the result of possible increased microbial activity in the warmer conditions within the brash (Asam et al. 2014). Nitrogen transformations within the stream channel, including nitrification, may be a further control streamwater inorganic N concentrations in headwater streams (Peterson et al. 2001).

The influence of landscape disturbance and recovery on macronutrient export was also apparent through two catchment land-use controls that indicate no recent disturbance: (i) proportion forested (in the 1960s–1970s) (i.e. that is unfelled) and (ii) proportion grassland and bog. One or both are significant negative controls in HY2012 on all macronutrient exports and on [DOC] and [SRP], but have no significant influence on export or concentration of any macronutrient in HY2013 (Table 4). The loss of influence of these two controls in HY2013 is attributed to new felling activities increasing macronutrient export and concentrations which buffering in undisturbed catchment areas is insufficient to counter.

The need to clear forest for wind farm construction was explained earlier. Thus, we classify forest-felling as a wind farm construction activity, although it is also undertaken at wind farm sites for timber harvesting at plantation maturity. Access tracks and turbine foundations are infrastructural requirements for wind energy generation, necessitating rock. To minimise the development C footprint, quarries (known as ‘borrow pits’) are often opened on site early in the construction period. They are usually later infilled with surplus excavated material (here predominantly peat) and capped with the overburden (soil and vegetation) removed when the pit was opened. In the catchment area studied, both borrow pits were restored. All infrastructural construction influenced the fluvial macronutrient response variables, to some extent and both positively and negatively. We now discuss these controls, considering first borrow pits.

Proportion borrow pits had a positive influence primarily on macronutrient export (DOC in both HYs, and POC and SRP export in HY2013) but also on [DOC] in HY2013, contrasting with a negative influence on [TON]. To the best of our knowledge, this is the first study to identify an effect on fluvial macronutrients of borrow pits on peatlands. Since our study methodology focused on understanding an integrated catchment response to multiple land uses, it was not possible to identify conclusively the processes by which borrow pits influence macronutrient export and concentration. Notwithstanding this knowledge gap, hypothesised processes were identified that might account for the effects of borrow pits identified in the MLRA. Similar to forest-felling, borrow pits are hypothesised to cause changes in hydrological pathways, resulting in enhanced runoff generation. Although borrow pits were restored through infilling with soil/peat, the removal and reinstatement of this material, no matter how carefully conducted, will likely change the functioning of the ground surface. Decomposition of organic material in the overburden may create a source of C and P which might be mobilised by enhanced runoff generation as the result of reduced vegetation cover (including the original forest cover removed by felling for borrow pit creation) causing lower interception and ET water losses. The negative control of borrow pits on [TON] is more difficult to explain. Several individual or complementary explanations are hypothesised: (i) enhanced labile C in borrow pits resulting from disturbance and restoration may lead to immobilisation of TON, (ii) conditions in borrow pits are conducive to any N occurring as ammonium-N rather than TON, and (iii) the construction and restoration of borrow pits may interrupt groundwater flow paths, resulting in lower inputs to streamwater of geological N (Holloway and Dahlgren 2002).

In summary, sub-catchments with borrow pits now have a new surface more important in generating macronutrient losses than catchments without borrow pits. Although borrow pits were restored before the end of construction, their influence was still apparent in HY2013 and might be expected to continue until the borrow pit-contents have equilibrated with the landscape hydrologically and as macronutrient sources. Both these require a functioning vegetation cover, as this influences C sequestration and water table dynamics, which could take several years to re-establish (Waldron et al. 2018). To identify more definitively the processes by which borrow pits may influence macronutrient concentration and export will require before-after-control-impact (BACI) studies comparing runoff and macronutrient flow pathways, concentrations and export in paired catchments in which the only land-use change is borrow pit construction. Even if such studies were conducted, care would be required in extending the results to other catchments as impacts are expected to depend on the borrow pit configuration and topographic setting. For example, the effect of borrow pit development and restoration on hydrological flow pathways might be hypothesised to be very different for a deep borrow pit located in the side of a slope compared to a shallow borrow pit in flat terrain.

The construction of tracks and turbines also requires excavation of soil, sometimes down to bedrock. Floating roads comprise aggregate on a geotextile layer placed on the peat surface and may cause peat subsidence over time (due to compression and possibly drainage). Both road types were used here and are predicted to enhance macronutrient loading, similar to borrow pits, due to changes in vegetation and surface soil hydrological conditions. Turbines are positioned along tracks and so track density and turbine density were correlated. However, to capture disturbance impact, a combined estimate of area impacted may be more accurate and was also explored in the MLRA. Whilst track and turbine density were shown here to positively influence [SRP] and its export, and occasionally [DOC], they do not appear consistently as a control on increasing fluvial macronutrient loadings. This may be due to the runoff management measures implemented at the site, such as settling ponds alongside tracks.

Although we infer that the disturbed new surface of borrow pits can enhance C and SRP export, we do not observe the catchment control of soil type (considered as % peat in the MLRA, see Table 2) to influence macronutrient export, which was unexpected. This may be because all catchments have > 71% peat cover (median 86%), so soil type is little differentiated. Of the other physiographic catchment characteristics considered, drainage density positively influences [TON]. This can be explained by the occurrence of more mineral-rich peaty gley and alluvial soils near river channels (Fig. 2b), suggesting these soil types (although not in the model) might influence macronutrient loading. The mineral fraction in gley soils has the capacity to mitigate SRP in subsurface runoff (Neal et al. 2003), so likely buffers [SRP], but insufficiently to counter the influence of construction disturbance on P loading.

The explanatory power of the predictor variables in the MLRA models varied between macronutrients and HY2012 and HY2013 (Table 4). Models often had high *R*^2^, but did not explain fully the field observations. The remaining unexplained variability in streamwater macronutrient concentration and export may relate to short-term and seasonal variations in weather and climate and long-term trends in atmospheric N deposition (Vuorenmaa et al. 2018). For example, enhanced streamwater [NO_3_–N] may occur for 2–3 years in upland UK catchments after severe summer drought (Reynolds et al. 1992), but such effects are difficult to disentangle from land-use activities in this study. Marked seasonality in macronutrient concentrations in temperate streamwaters may also confound interpretation based on catchment descriptors. [DOC] typically peaks in late summer/autumn from flushing of DOC accumulated in organic soils by microbial activity in warmer summer temperatures (e.g. Waldron et al. 2009), whilst peak [TON] normally occurs in winter when plant activity and N uptake is low (e.g. Smart et al. 2005). Both [DOC] and [TON] are influenced by primary production and uptake in the catchment, and antecedent weather conditions influencing flow pathways and soil moisture.

### Lag times of macronutrient response to wind farm development activities and recovery

The long-term [SRP] time series at the catchment exit (WL15_1, Fig. 1) revealed a rapid response to development activities. [SRP] increased from typically < 40 to ~ 80 µg P L^−1^ 6 months after extension phase 2 commenced. Elevated [SRP] is maintained for ~ 1.5 years, before concentrations begin to decline. A switch to seasonal [SRP] patterns peaking in summer is apparent, as also reported in peatland drainage after conifer-felling in Ireland (Cummins and Farrell 2003a), most likely due to enhanced decomposition and release of P from brash material remaining on site. In contrast to [SRP], the time lag between forest-felling and increased [TON] is > 1 year.

**Fig. 6:**
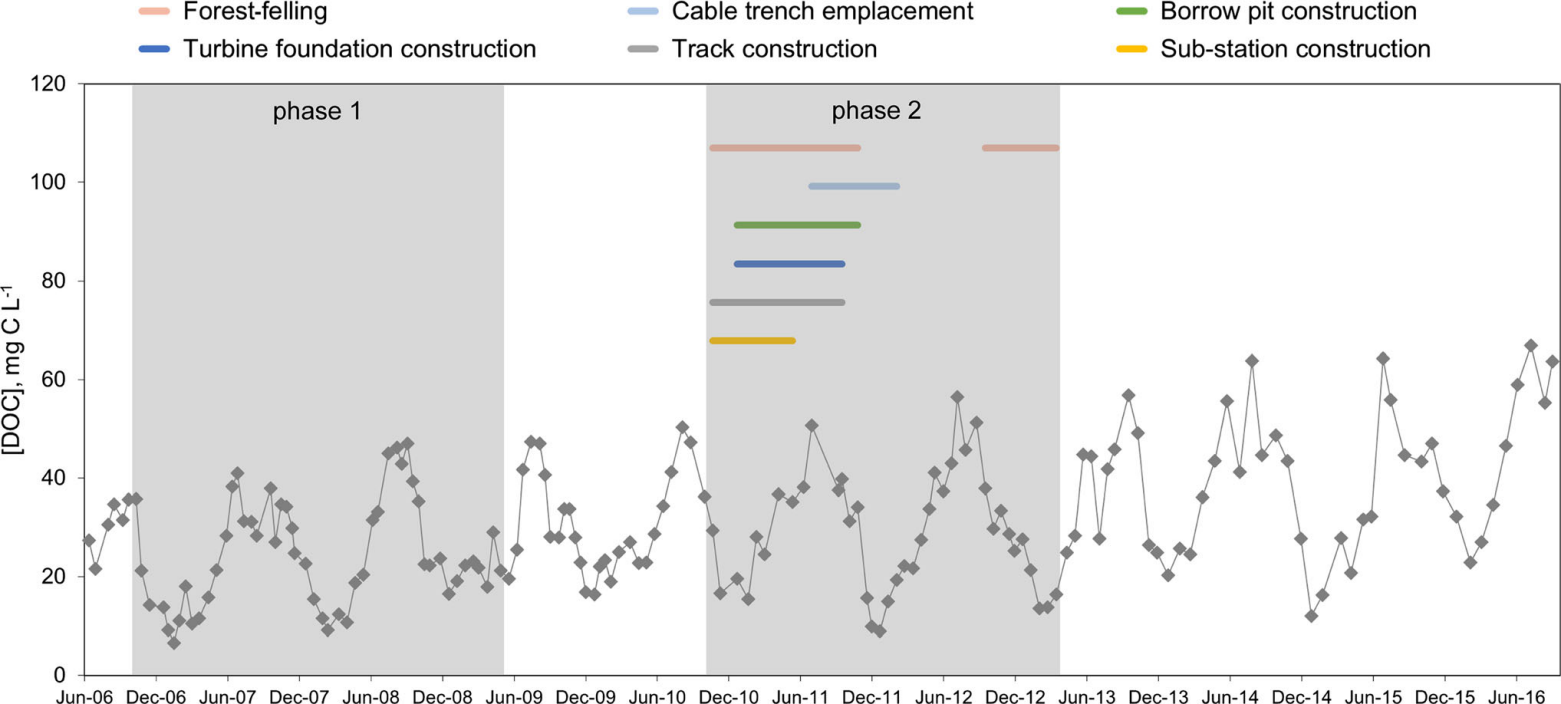
Dissolved organic carbon (DOC) concentrations measured at the outlet of catchment WL15 (sampling point 15_1, see Fig. 2a for location), July 2006–September 2016. The boxes show the timing of phases 1–2 of wind farm development, whilst the colour bars depict the timing of different activities during phase 2

[DOC] response has a similar time lag of ~ 1.5 years: the WL15_1 [DOC] long-term time series indicates increasing annual maximum [DOC] after extension activities commence, from ≤ 50 to 56–67 mg C L^−1^ from July 2012 (Fig. 6). A similar pattern of increased annual maximum [DOC] superimposed on the existing seasonal pattern in drainage has been reported elsewhere following forest-felling on peatlands and in boreal catchments, associated with enhanced biological productivity in warmer summer temperatures (Cummins and Farrell 2003b; Schelker et al. 2012). As with [SRP], [DOC] appears enhanced for the remainder of the long-term record, but it is uncertain whether this is the result of other drivers, such as higher soil temperature or changes in soil solution chemistry due to decreased sulphate deposition (Sawicka et al. 2017) or N accumulation or flow (Erlandsson et al. 2008), acting separately or in combination with land-use change.

### Implications for wind farm development and forest-felling on peatlands

Pressure for land-use change in peatlands is increasing, for example due to population expansion and development of wind farms. Markbygden wind farm, Europe’s largest onshore wind farm is under development in northern Sweden, comprising up to 1101 turbines and 4000 MW generating capacity, increasing Sweden’s renewable generating capacity by 12.5% when completed (Svevind 2019). Peatlands can be already impacted: degradation is estimated to affect almost half the peatland area of Great Britain, and in England < 2% of the ~ 3500 km^2^ of blanket bog is assessed to be undamaged (JNCC 2011). Peatland disturbance by forest-felling has been shown by many studies to increase macronutrient concentrations and exports to receiving aquatic systems (e.g. Cummins and Farrell 2003a, b; Nieminen et al. 2017). Now, we document the construction of wind farm infrastructure on peatlands as another driver of fluvial macronutrient impact.

During the period of the wind farm extension, the WFD status based on [SRP] was classified as “poor” in five sub-catchments (15_4, 15_5, 15_8, 15_15, 15_20) and “bad” in one (15_7). These sub-catchments were all small (< 0.85 km^2^ area) and were generally the most disturbed (all > 45% forest-felling and including all sub-catchments > 1% borrow pit). The river water [SRP] status improved downstream at the catchment exit (15_1) during the extension construction to “moderate”/“poor”, attributed to processes such as instream dilution, biological processing and adsorption to riverine sediment (Withers and Jarvie 2008), and subsequently recovered to “moderate” and occasionally “good” status. Whilst the WFD EQS apply to “main waterbody” catchments larger than here, if pre-disturbance [SRP] was close to a threshold, then forest-felling and/or wind farm construction could cause a downgrade in status or contribute to failure to achieve a required WFD classification improvement. Although not quantified here, river water dissolved organic N and P might increase too following disturbance such as forest-felling (Schelker et al. 2016). Thus, to fully understand response to wind farm construction, particularly in sensitive waters, all macronutrient pools may require characterisation.

The Scottish Government (2017) considers areas identified for onshore wind farms must be “suitable for use in perpetuity”. If WFD compliance is necessary, other measures to minimise impact on and restore fluvial macronutrient status will be needed for wind farm developments such as these. Good practice guidance is widely available in the UK (e.g. wind farm construction (Joint publication 2015), floating roads on peat (SNH and FCE 2010), track construction (SNH 2013), forestry activities (Forestry Commission 2017)), and in other countries (e.g. France; ONF 2017). Particularly relevant measures for minimising increased macronutrient fluvial loadings from these and other sources include the following:

- Confining disturbance to small areas away from sensitive water courses
- Avoiding disturbing sloped areas
- Adopting runoff and sediment management measures
- Following good practice for forests and water (e.g. keeping streams and buffer areas clear of brash as far as practicable)
- In boreal forests, timing harvesting on frozen ground during winter to decrease soil disturbance (Nieminen et al. 2017)
- Appropriate felling waste management. Whilst brash mats are commonly recommended during felling to minimise soil erosion due to heavy machinery (Moffat et al. 2006), if peatland restoration or maintaining low-nutrient waterbodies are priorities, removing felling waste should be considered.
- Avoid or reduce peat displacement from the development of borrow pits, and justify the need, use and location of borrow pits (consistent with Scottish Planning Policy; Scottish Government 2014).

The wind farm construction guidance (Joint publication 2015) asks if borrow pits proposed are in “suitable locations (i.e. close to proposed construction routes, to minimise haul distances)”. Where sites have significant overburden (soil depth), the developer should “consider the economic viability and practicality (construction logistics and transport impacts) of importing aggregate” (Scottish Renewables and SEPA 2012). Sourcing aggregate from outside the site of wind farm infrastructure in non-peat areas may also be an important action to minimise fluvial impact. The effect on the C footprint of a wind farm of sourcing aggregate on a peatland site versus the transport emissions of importing aggregate from non-peat areas would require evaluation for each individual wind farm site.

Our findings that wind farm infrastructure construction is associated with increased fluvial macronutrient concentration and export also has implications for the future development of existing wind farms on peatlands. The industry is now looking to replace current turbines with larger turbines (known as “re-powering”), particularly at sites approaching the end of the lifetime granted under the original planning permission (Waldron et al. 2018). The use of existing tracks and turbine bases for re-powering is unlikely to have a major effect on aquatic nutrients, if runoff management measures are in place. However, if the construction of new foundations requires new aggregate, then opening up new borrow pits or revisiting old ones on peatlands could result in increased C and P fluvial export.

## Conclusion

This is the first study to investigate which specific wind farm development activities on peatlands affect fluvial macronutrient concentration and export. Forest-felling, borrow pits, and to a lesser extent turbine base and track construction were the activities identified as significant drivers of fluvial macronutrient loading. River water [DOC] and [SRP] increased significantly from upstream to downstream in the catchment during wind farm development, although export rates were similar. Streamwater SRP status was lowest during wind farm development in headwater sub-catchments (< 0.85 km^2^ area) with the greatest proportion of area disturbed by forest-felling and borrow pit construction. A deterioration in SRP status was also detected at the catchment outlet (~ 12 km^2^), though to a lesser extent. The impacts on fluvial macronutrients of wind farm development on peatlands appear to be greatest in small catchments proportionally most affected by construction activities and associated forest-felling. The effects on SRP and DOC concentration propagated downstream (in this study a distance of a few km), but intermediate attenuation occurred, attributed to dilution and instream processing. Whilst further attenuation is anticipated downstream, any increase in [SRP] and [DOC] could be of concern for oligotrophic waterbodies and surface drinking water supplies. The main practical implications arising from this research for wind farm development on peatlands in order to minimise macronutrient loss to rivers are (i) limit within individual catchments the proportion disturbance by both infrastructure construction and forest-felling activities (at least < 45%); (ii) phase wind farm construction and forest-felling for timber harvesting by at least ~ 2 years; and (iii) consider fluvial macronutrient losses from land, as well as construction logistics and transport impacts, in proposing borrow pit locations.

## Electronic supplementary material

Below is the link to the electronic supplementary material.

Supplementary material 1 (PDF 1025 kb)
