# Supplementary material for: Wind farm development on peatlands increases fluvial macronutrient loading
Source: Ambio. 2019 May 28;49(2):442–59. doi: 10.1007/s13280-019-01200-2 (PMC6965044; doi:10.1007/s13280-019-01200-2)
Supplement: Supplementary file 1 — Supplementary material 1 (PDF 1025 kb) [file 13280_2019_1200_MOESM1_ESM.pdf]

Ambio

Electronic Supplementary Material

This supplementary material has not been peer reviewed.

Title: **Wind farm development on peatlands increases fluvial macronutrient loading**

Authors: Kate Heal, Antony Phin, Susan Waldron, Hugh Flowers, Patricia Bruneau, Andrew Coupar, Andrew Cundill

Fig. S1. Mean daily flow at 15_1 plotted on the flow duration curve at 15_1 for hydrological years 2012 and 2013 showing that the streamwater sampling programme was successful in targeting a range of flows. Discharge (Q) is on a log_{10} scale.

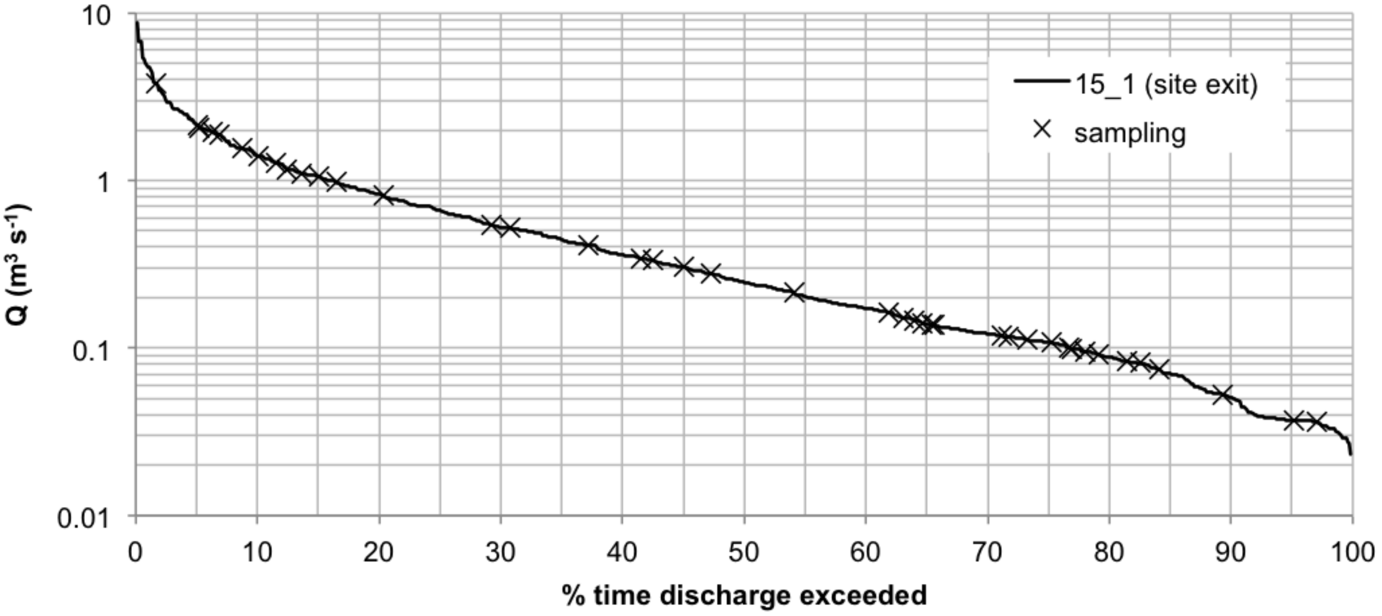

S1. Methods for collection and analysis of water samples

Samples for carbon (C) species analysis were collected in 1 L Nalgene® bottles and caps, pre-cleaned in 2% phosphate-free Decon® solution and rinsed with deionised water. Samples for soluble reactive phosphorus (SRP) and total oxidised nitrogen (TON, NO_3^- plus NO_2^-) analysis were collected in sterile 60 mL vials. Gloves were worn during sampling and all sampling containers and tops were rinsed three times with streamwater before sample collection. Samples were stored in a cool box with ice packs until return to the laboratory, where they were stored at 4°C until analysis.

A known volume (~0.5 L) of the water sample for C species analysis was vacuum filtered within 48 h of collection using pre-ashed (450°C, 8 h) Whatman® GF/F 0.7 µm filters. Particulate organic carbon (POC) was determined by weight difference of the residue on the filter before and after heating to 375°C (Ball 1964), assuming that carbon made up 58% of the particulate organic matter lost (van Bemmelen 1890). POC values determined in samples from October 2011 to June 2012 should be regarded as minimum values due to a furnace fault. For quantification of dissolved organic carbon (DOC), a 60 mL aliquot of the filtrate was acidified to pH 3.9 using 0.05 M H_2SO_4

then placed in an ultrasonic bath to degas any dissolved CO_2, before analysis within 3-6 months using a Thermolux total carbon analyser.

Water samples for P and N species analysis were syringe-filtered with Whatman® 47 mm diameter 0.2 µm nylon membrane filters and stored in 50 mL HDPE containers at 4°C. All glassware and filter assemblies used in sample preparation and analysis were soaked in 2% phosphate free Decon® solution overnight. Determination of SRP and TON concentrations by colorimetric methods using a Bran+Luebbe® Autoanalyzer 3 was usually carried out within 24 h of sample collection. SRP was quantified as phosphate $[PO_4]^{3-}$ at 660 nm using an ammonium molybdate-ascorbic acid method (Murphy and Riley 1962; Method No. G-103-93 Rev. 1 with increased $[H_2SO_4]$ to increase sensitivity). Due to the high dissolved organic matter content of the samples, [SRP] was corrected for colour interference by analysing samples without the ammonium molybdate reagent and subtracting the absorbance. Colour interference was estimated to account for 1-14 µg P L^{-1} across the long term monitoring catchments (Murray 2012). TON was determined at 550 nm using a hydrazine reduction method to form a pink azo-dye (Mullin and Riley 1955; Method No. G-109-94 Rev. 3). TON was assumed to comprise predominantly NO_3^- since previous streamwater sampling at WL15_1 (Murray 2012) had found that $[NO_2^-]$ was negligible (<1 to 16 µg NO_2-N L^{-1}, sampled on 62 occasions 2006-2010) and was 1-2 orders of magnitude lower than $[NO_3^-]$.

Instruments were calibrated using a range of standards of appropriate concentrations and standards were analysed during runs to check for instrument drift. Analytical runs for DOC, POC, SRP and TON were accompanied by 2-3 procedural blanks, consisting of deionised water prepared in the same way as the samples. All concentrations are reported as blank corrected. An additional field blank was measured on three occasions, where a 1 L Nalgene® container was filled with deionised water and taken to site, to account for any cross contamination that might have occurred through reusing containers. Mean limits of detection were 6.3 µg P L^{-1} and 0.08 mg N L^{-1}. Since [SRP] in the 10-year monitoring programme was determined using different instruments at the Universities of Edinburgh and Glasgow, an inter-laboratory comparison was conducted which showed no significant difference in [SRP] between laboratories.

Alkalinity was determined on 11 unfiltered water samples from each sampling point collected from November 2011 to November 2012. A manual Gran titration method was used with 0.01 M HCl and a calibrated pH meter (British Standards Institution 1996) until all buffering components were consumed, as recommended for upland surface waters (Reynolds and Neal 1987).

S2. Flow and export estimates

Methods for estimating flows and macronutrient exports are summarised here, with further details in Phin (2016). Flows were estimated by deploying an area-velocity flow logger (Isco 4150) to record stream depth (via pressure transducer) and velocity (via Doppler ultrasound method) every 15 min for 2-6 months at four representative locations within the catchment (Fig. S2). Relationships between the flow measured at these four locations and at the nearest SEPA gauging station at Newmilns (~6 km to the south) were used in conjunction with scaled sub-catchment area (Table S1) to provide a continuous estimate of flow at each streamwater sampling point during the study period. The relationship between flows measured at location 1 (15_3) and at the SEPA gauging station is shown in Fig. S3.

Fig. S2 Schematic showing the representative flow logging locations (numbered 1 to 4 in boxes) across the WL15 catchment in relation to the streamwater sampling points and also the times of deployment of the Isco area-velocity logger at each location The R^2 values and nature of the relationships between Location 1 and SEPA Newmilns gauging station and between Locations 2-4 and Location 1 are also shown.

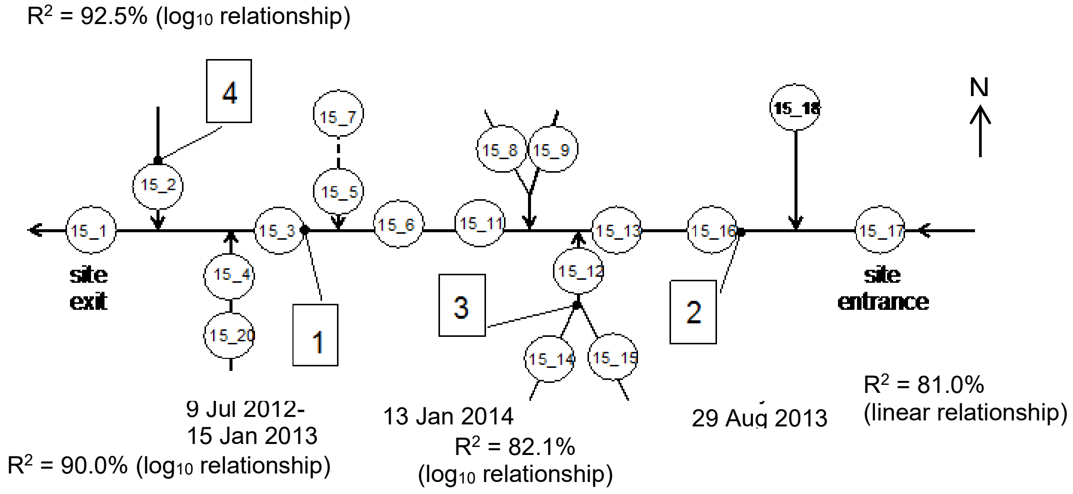

Fig. S3 Relationship between flow estimated by area-velocity method using an Isco logger at 15_3 and the SEPA Newmilns river gauging station every 15 min July 2012 to March 2013

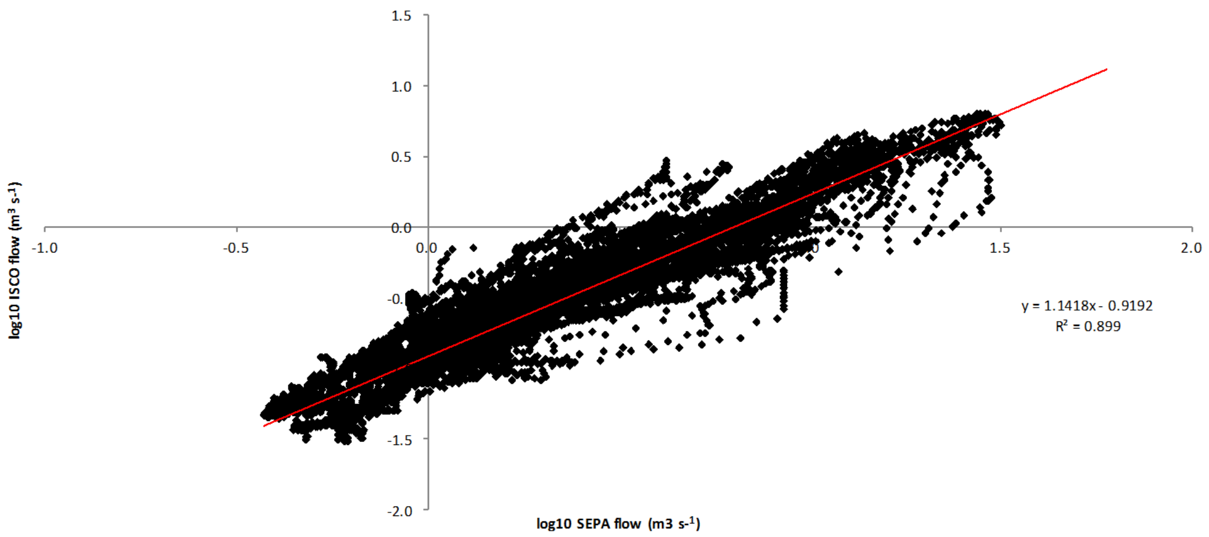

Mass exports of DOC, POC, SRP and TON were estimated for all streamwater sampling locations using the 15 min flow estimates and 3-weekly concentration data. Since there were no significant relationships between concentration and flow which would allow an extrapolation method to be used, an interpolation method was used (method 5 in Walling and Webb 1985) which is suitable for datasets with monthly concentration measurements (Johnes 2007). In this method the export for each sampling day in HY2012 and HY2013 was calculated from mean daily flow and concentration. As recommended for datasets with high frequency flow data but relatively few concentration data, a ratio estimator was applied to the mean of the daily exports ($\overline{CQ}$) to account for covariance of export and flow (Quilbé et al. 2006) (Eq. S1). The corrected mean daily export (E) was then multiplied by 365 days and divided by the area of each sub-catchment to provide estimates of export in mass m^{-2} y^{-1}.

Equation S1 $$E = \overline{CQ}\left[\frac{\mu}{\bar{Q}}\right].n\left(\frac{1+\frac{1}{n_d}\frac{S_{CQ}}{\overline{CQ}\,\bar{Q}}}{1+\frac{1}{n_d}\frac{S_{Q^2}}{\bar{Q}^2}}\right)$$

Where μ is the mean discharge over the period of interest, $\bar{Q}$ is the mean of the mean daily flow over the sampling days in the period, n is the number of days in the period,

$S_{CQ} = \frac{1}{n_d - 1}\left(\sum_{i=1}^{n} A_i C_i Q_i - n_d\, \overline{CQ}\, \bar{Q}\right)$; and $S_{Q^2} = \frac{1}{n_d - 1}\left(\sum_{i=1}^{n} A_i Q_i - n_d\, \bar{Q}^2\right)$

A_i indicates availability of concentration data (1 if available, 0 if not), C_i is the concentration on sampling day i, Q_i is the mean flow on day i, and n_d is the total number of days where concentration data was available over the time period of export calculation.

Table S1 Catchment areas of the WL15 catchment streamwater sampling points as a proportion of flow logging location catchment areas

**Flow logger location**	**Area (km^2)**	**Proportion of catchment**			
		Location 1	Location 2	Location 3	Location 4
Location 1 (15_3)	8.66	1.00			
15_1	11.45	1.32			
15_6	8.19	0.95			
15_11	7.64	0.88			
15_13	3.92	0.45			
Location 2 (15_16)	2.70		1.00		
15_18	1.16		0.43		
15_17	0.50		0.19		
Location 3 (c.500 m downstream from 15_14/15) confluence	1.81			1.00	
15_12	2.69			1.49	
15_15	0.83			0.46	
15_14	0.82			0.46	
15_4	0.76			0.42	
15_20	0.41			0.23	
Location 4 (15_2)	0.41				1.00
15_9	0.53				1.31
15_8	0.26				0.64
15_5	0.16				0.39
15_7	0.03				0.08

Table S2 Physiographic characteristics of the sub-catchment area draining to each streamwater sampling point. % totals for soil type and drainage class do not equal 100 % for some sub-catchments due to rounding

Sampling point	Soil type (%)				Drainage class (%)					Stream length (km)	Drainage density (km km^{-2})	Mean slope ± s.d. (°)	Max. slope (°)
	Peat	Peaty gley	Podzol /gley	Alluvial	Very poor	Poor	Poor/ free	Imperfect	Free				
15_1	78	11	7	4	81	11	1	2	5	30	2.7	2.9 ± 2.2	20
15_2	71	19	10	0	71	19	0	0	10	1.5	3.7	3.1 ± 1.9	11
15_3	79	11	6	4	83	11	1	1	5	23	2.7	2.9 ± 2.1	20
15_4	85	4	11	0	92	4	2	2	1	1.5	2.0	2.8 ± 1.5	9
15_5	82	18	0	0	82	18	0	0	0	0.6	3.9	2.9 ± 2.1	9
15_6	81	9	5	4	86	9	1	0	5	21	2.6	2.9 ± 2.1	20
15_7	100	0	0	0	100	0	0	0	0	0.1	2.9	2.4 ± 2.0	7
15_8	73	11	16	0	89	11	0	0	0	0.8	2.9	2.3 ± 1.9	10
15_9	82	8	10	0	92	8	0	0	0	1.0	1.8	2.0 ± 1.6	9
15_11	83	8	6	4	87	8	1	0	4	20	2.6	2.8 ± 1.9	15
15_12	80	10	6	4	84	10	2	0	4	9.0	3.3	3.0 ± 2.0	13
15_13	86	5	4	4	91	5	0	0	4	8.2	2.1	2.8 ± 1.9	15
15_14	86	1	13	1	98	1	0	0	1	4.1	5.0	2.6 ± 1.7	12
15_15	86	9	5	0	86	9	5	0	0	2.1	2.5	3.1 ± 2.2	13
15_16	92	2	6	0	98	2	0	0	0	4.7	1.7	2.8 ± 1.8	11
15_17	87	0	13	0	100	0	0	0	0	1.0	1.9	3.1 ± 1.8	10
15_18	99	1	0	0	99	1	0	0	0	0.8	0.7	2.7 ± 1.9	11
15_20	93	3	4	0	94	3	4	0	0	0.7	1.8	3.1 ± 1.5	9

S3. GIS datasets and multiple linear regression analysis

UK Ordnance Survey Land-Form PROFILE DTM data at 1:10 000 scale were used to delineate catchment area and stream networks (using the fill, flow direction and flow accumulation tools) and slope. James Hutton Institute soils data (derived from the Soil Survey of Scotland 1: 25 000 scale maps) and land-use data were used to describe the soils (and their drainage classes) and land use. Forest plantation area was determined using a shapefile obtained from the Forestry Commission National Forest Inventory (2011). Areas and the year of forest-felling were delineated from information received from Forest Research in October 2014, and verified and added to from site observations. An additional information layer was added from site observations and the as-built wind farm layout.

Catchment selection for multiple linear regression analysis (MLRA) aimed to maximise the use of the data collected to create understanding of the drivers of macronutrient response to land-use activities, whilst minimising the influence of nested sampling points. Thus the selected catchments included not only the nine independent headwater catchments, but also four downstream sampling points representing additional land-use influences and/or substantial increases in catchment area bringing in new material. All nine independent headwater catchments - 15_2, 15_7, 15_8, 15_9, 15_14, 15_15, 15_17, 15_18, 15_20 - were included as these sampling points represent different primary wind farm development activities alone and in combination, including different ages of forest-felling, and/or showed different macronutrient responses. For example, catchments 15_14 and 15_15 have very similar physiography and both contain turbines and tracks, but 47% of the area of 15_15 has been affected by forest-felling compared to 1% in 15_14. 15_18 represents the influence of the sub-station, in contrast to adjacent headwater catchment 15_17, and also has the highest % cover of grassland & bog. 15_8 and 15_9 are also similar and adjacent, but 15_8 has been primarily affected by forest-felling, whilst 15_9 contains more turbines in addition to containing forest-felling. Although 15_7, 15_8 and 15_9 are adjacent and have all been affected by the most recent forest-felling, their macronutrient responses are very different ([DOC] and [SRP] at 15_7 are much higher, whilst [POC] is particularly high at 15_7 and 15_8). Therefore all these three catchments were included in the regression analysis to help understand the drivers of the observed variability in response.

Of the downstream sampling points within which the headwater catchments are nested, 15_4, 15-5, 15_12 and 15_13 were selected for inclusion in MLRA for the following reasons. Although downstream of 15_7, 15_5 represents a >400% increase in catchment area and also contains the greatest influence of a borrow pit, compared to any of the other sampling points. 15_4, downstream of 15_20, accounts for an 85% increase in catchment area, and brings in areas of intact forest plantation compared to 15_20 where 96% of the catchment area comprises forest felled at different times in 2006 and 2010. 15_12 and 15_13 account for increases in catchment area of 63% and 45%, respectively, compared to their nearest upstream sampling point and also bring in influences of grassland & bog land cover and a wider range of soil types.

The median annual concentrations and total annual exports per unit area of DOC, POC, SRP and TON for the selected WL15 sub-catchments were used as the response variables in the MLRA. The median was used for the annual concentrations as this was less affected by extreme values. Separate analyses were conducted for HY2012 and HY2013 to consider the timing of wind farm development controls. n=13 for HY2012 MRLA and n=12 for HY2012 MLRA due to cessation of sampling at 15_18 in May 2013 because of changed hydrological conditions as the result of drainage diversion. The possible predictor variables included all those listed in Tables 1 and S2, plus a combined wind farm infrastructure disturbance variable (track density + turbine density).

Multiple linear regression analysis (MLRA) was conducted in the following steps:

1. Checked for normality of the response variables and all possible predictor variables by inspecting normal probability plots and the outcome of Anderson-Darling tests at 95% confidence. All response variables required transformation ($\log_{10}$ or 1/x transformations) to achieve normal distributions. Of the possible predictor variables requiring transformation, some (catchment area, stream length, turbine density, track density + turbine density) were transformed successfully, whilst for two predictor variables (proportion borrow pit and distance to nearest disturbance) normal distributions could not be achieved even after trialling many transformations.
2. Checked for correlation between possible predictor variables using Pearson product moment correlation coefficients. Where possible predictor variables were significantly correlated ($p<0.05$), only one was selected for inclusion in the MLRA. For example, proportion peat was significantly correlated with proportion peaty gley, proportion podzol and proportion of different soil drainage classes, so only proportion peat was

selected. Process understanding was then used to select the final predictor variables as explained in Table 2.

3. Conducted best subsets regression to identify potential models for each response variable, aiming to maximise R^2 (adjusted), minimise the number of predictor variables, minimise the S value, and obtain Cp values similar to the number of variables.
4. The shortlisted potential models were then examined individually using stepwise regression to select the model for each response variable. After each predictor variable was added it was checked that: i) the R^2 (adjusted) had increased, ii) the variance inflation factor (VIF) was <5 to avoid multicollinearity effects, and iii) $p<0.05$ for each variable. Residuals were also checked for normality and plots of residuals against fitted values were inspected to check that residuals were randomly dispersed. In the selected models the regression coefficients were standardised (by subtracting the mean and dividing by the standard deviation) to make it easier to identify the relative importance of different variables (expressed in different units).

Three sets of MLRA were conducted to ensure that all possible dimensions of wind farm infrastructure were considered. The first MLRA (model 1) was conducted including track density amongst the predictor variables and not turbine density because these two variables are significantly positively correlated. Re-running the MLRA (model 2) including turbine density rather than track density in the predictor variables yielded some differences in model selection. Therefore the third run of the MLRA (model 3) included a combined wind farm infrastructure disturbance variable (track density + turbine density). The outputs from the three MLRA models are summarised in Table S3, highlighting the significant positive and negative controls on each response variable for each hydrological year (HY2012 and HY2013).

Table S3 Summary of significant controls on annual (a) exports (g m^{-2}) and (b) median concentrations (mg L^{-1} apart from µg L^{-1} for SRP) of DOC, POC, SRP and TON for HY2012 and HY2013 in the three sets of multiple linear regression analyses (MLRA) conducted. POC modelled for HY2013 only due to possible under-estimation in HY2012 (see S1). M1, M2, M3 = models including track density only, turbine density only, track density + turbine density, respectively. -12, -13 = HY2012 and HY2013. +/- signs indicate significant positive/negative controls. Numbers in parentheses indicate % R^2 (adj) accounted for in the model. Grey blocks highlight consistent controls across model sets. Black blocks indicate not included in the MLRA. Distance to nearest disturbance not shown since it was not significant in any selected MLRA models.

(a) exports	Catchment physiographic controls					Wind farm construction controls						R^2 (adj) (%)
MLRA model	Mean slope	Drainage density	Prop. peat	Prop. forest	Prop. grassland & bog	Track density	Turbine density	Track density + Turbine density	Prop. forest-felled	Prop. forest-felled > 1 year	Prop. borrow pit	
DOC M1-12				- (16.9)	- (17.3)					- (21.9)	+ (30.0)	86.1
DOC M2-12				- (16.9)	- (17.3)					- (21.9)	+ (30.0)	86.1
DOC M3-12				- (16.9)	- (17.3)					- (21.9)	+ (30.0)	86.1
DOC M1-13									+ (23.4)	- (28.6)	+ (35.2)	87.2
DOC M2-13									+ (23.4)	- (28.6)	+ (35.2)	87.2
DOC M3-13									+ (23.4)	- (28.6)	+ (35.2)	87.2
POC M1-13									+ (57.0)	- (10.9)	+ (17.7)	85.5
POC M2-13									+ (57.0)	- (10.9)	+ (17.7)	85.5
POC M3-13									+ (57.0)	- (10.9)	+ (17.7)	85.5
SRP M1-12	+ (5.0)		+ (2.7)	- (26.6)	- (40.6)					- (1.1)	+ (11.8)	87.7
SRP M2-12				- (24.5)			+ (52.2)					76.7
SRP M3-12		+ (10.4)		- (26.1)				+ (42.7)				79.1
SRP M1-13									+ (55.5)	- (9.4)	+ (22.3)	87.1
SRP M2-13									+ (55.5)	- (9.4)	+ (22.3)	87.1
SRP M3-13									+ (55.5)	- (9.4)	+ (22.3)	87.1
TON M1-12					- (17.4)							17.4
TON M2-12					- (17.4)							17.4
TON M3-12					- (17.4)							17.4
TON M1-13									+ (51.4)			51.4
TON M2-13									+ (51.4)			51.4
TON M3-13									+ (51.4)			51.4

(b) Concentrations	Catchment physiographic controls					Wind farm construction controls						R^2 (adj) (%)
MLRA model	Mean slope	Drainage density	Prop. peat	Prop. forest	Prop. grassland & bog	Track density	Turbine density	Track density + Turbine density	Prop. forest-felled	Prop. forest-felled > 1 year	Prop. borrow pit	
DOC M1-12		+ (2.6)	+ (4.8)	- (17.9)	- (41.7)							67.1
DOC M2-12	+ (20.7)						+ (50.5)					71.2
DOC M3-12	+ (12.0)				- (41.7)			+ (18.6)				72.4
DOC M1-13			+ (16.1)		- (21.8)	- (24.0)				- (30.6)		92.4
DOC M2-13	+ (21.6)		- (24.3)	- (11.5)			+ (24.8)					82.3
DOC M3-13									+ (38.3)	- (26.4)	+ (10.9)	75.7
POC M1-13									+ (70.8)			70.8
POC M2-13									+ (70.8)			70.8
POC M3-13									+ (70.8)			70.8
SRP M1-12	+ (16.9)			- (17.3)		+ (43.1)						77.4
SRP M2-12							+ (39.0)			+ (21.5)	- (17.0)	77.5
SRP M3-12	+ (27.4)			- (6.7)				+ (54.0)				88.1
SRP M1-13			+ (17.5)						+ (57.3)			74.8
SRP M2-13							+ (58.4)		+ (24.8)			89.1
SRP M3-13								+ (69.4)	+ (21.6)			91.0
TON M1-12		+ (10.7)								+ (18.8)	- (50.3)	79.8
TON M2-12		+ (10.7)								+ (18.8)	- (50.3)	79.8
TON M3-12		+ (10.7)								+ (18.8)	- (50.3)	79.8
TON M1-13						+ (11.0)				+ (45.5)	- (26.9)	83.3
TON M2-13	- (14.8)	+ (4.0)								+ (45.5)	- (26.9)	91.1
TON M3-13	- (14.8)	+ (4.0)								+ (45.5)	- (26.9)	91.1

References

Ball, D.F. 1964. Loss-on-ignition as an estimate of organic matter and organic carbon in non-calcareous soils. *Journal of Soil Science* 15: 84–92.

British Standards Institution (BSI). 1996. Water quality - Determination of alkalinity - Part 1: Determination of total and composite alkalinity. BSI, BS EN ISO 9963-1:1996, London.

Johnes, P.J. 2007. Uncertainties in annual riverine phosphorus load estimation: Impact of load estimation methodology, sampling frequency, baseflow index and catchment population density. *Journal of Hydrology* 332: 241–258.

Mullin, J.B., and J.P. Riley. 1955. The spectrophotometric determination of nitrate in natural water with particular reference to sea water. *Analytica Chimica Acta* 12: 464–480.

Murphy, J., and J.P. Riley. 1962. A modified single solution method for the determination of phosphate in natural waters. *Analytica Chimica Acta* 27: 31–36.

Phin, A. 2016. Quantifying Impacts of Peatland-based Windfarm Development on Aquatic Carbon and Nutrient Exports. PhD thesis. Edinburgh, Scotland: University of Edinburgh.

Quilbé, R., A.N. Rousseau, M. Duchemin, A. Poulin, G. Gangbazo, and J.-P. Villeneuve. 2006. Selecting a calculation method to estimate sediment and nutrient loads in streams: Application to the Beaurivage River (Québec, Canada). *Journal of Hydrology* 326: 295–310.

Reynolds B., and C. Neal. 1987. A comment on the use of acidimetric titrations for the estimation of the alkalinity and bicarbonate content of acid upland surface waters. *Science of the Total Environment* 65: 155–161.

van Bemmelen, P. 1890. Über die Bestimmung des Wassers, des Humus, des Schwefels, der in den colloïdalen Silikaten gebundenen Kieselsäure, des Mangans u. s. w. im Ackerboden. *Die Landwirthschaftlichen Versuchs-Stationen* 37: 279–290 (in German).

Walling, D.E., and B.W. Webb. 1985. Estimating the discharge of contaminants to coastal waters by rivers: some cautionary comments. *Marine Pollution Bulletin* 16: 488–492.
